# Acute stress alters recognition memory and AMPA/NMDA receptor subunits in a sex-dependent manner

**DOI:** 10.1016/j.ynstr.2023.100545

**Published:** 2023-05-26

**Authors:** Sebastiano A. Torrisi, Silvia Rizzo, Samuele Laudani, Alessandro Ieraci, Filippo Drago, Gian Marco Leggio

**Affiliations:** a Department of Biomedical and Biotechnological Sciences, University of Catania, Catania, Italy; b Department of Theoretical and Applied Sciences, eCampus University, 22060, Novedrate, CO, Italy

**Keywords:** Stress, Sex, Memory, Glutamate, NMDA, AMPA

## Abstract

Several studies have consistently reported a detrimental effect of chronic stress on recognition memory. However, the effects of acute stress on this cognitive ability have been poorly investigated. Moreover, despite well-documented sex differences in recognition memory observed in clinical studies, most of the preclinical studies in this field of research have been carried out by using solely male rodents. Here we tested the hypothesis that acute stress could affect the consolidation of different types of recognition memory in a sex-dependent manner. For this purpose, male and female C57BL6/J mice were exposed to 2-h of restrain stress immediately after the training session of both the novel object recognition (NOR) test and novel object location (NOL) tasks. Acute restraint stress did not affect memory performance of male and female mice, after a 4-h delay between the training session and the test phase of both tasks. By contrast, acute restraint stress altered memory performance in a sex-dependent manner, after a 24-h delay. While stressed mice of both sexes were impaired in the NOL test, only male stressed mice were impaired in the NOR test. Because ionotropic glutamate receptors-mediated neurotransmission is essential for shaping recognition memory, we further tested the hypothesis that post training acute stress could induce sex-dependent transcriptional changes of ionotropic glutamate receptor subunits in the dorsal hippocampus. We uncovered that acute stress induced sex-, time- and type of memory-dependent transcriptional changes of N-methyl-D-aspartate (NMDA) and α-amino-3-hydroxy-5-methyl-4-isoxazolepropionic acid (AMPA) receptor subunits. These findings suggest that the effect of acute stress on recognition memory can be strongly biased by multiple factors including sex. These findings also indicate that the same stress-induced memory impairment observed in both sexes can be triggered by different sex-dependent molecular mechanisms. At the therapeutic level, this should not be overlooked in the context of personalized and targeted treatments.

## Highlights

• Acute stress alters recognition memory in a sex-dependent manner.
• Acutestress-induced memory impairment is associated with sex-dependent transcriptional alterations of NMDA/AMPA receptors.
• Acute stress induces immediate sex-dependent transcriptional alterations of NMDA/AMPA receptors.

## 1 Introduction

Stress can trigger divergent effects on cognitive functions including learning and memory (Luksys and Sandi, 2011). These effects are expected to depend on multifactorial interactions (Sandi, 2013). However, despite plenty of studies have produced key findings concerning the neurobiological mechanism underlying the influence of stress on different types of memory, multiple contrasting findings push towards the need for additional investigation. Recognition memory is a cognitive function that allow to recognize and make judgments about stimuli or events encountered before (Barker and Warburton, 2011). The effect of stress on recognition memory is still controversial even though it has been extensively studied by using well-established rodent behavioral tasks (Moreira et al., 2016). In this respect, there exists the general concept that chronic stress exerts deleterious effects on recognition memory (Sandi and Pinelo-Nava, 2007). However, this concept has been subverted by studies assessing the influence of chronic stress in both male and female rodents (Luine et al., 2017). It was reported indeed that 6-h restraint stress for 21 days impaired the object recognition memory of male rats but not that of female rats (Beck and Luine, 2002). The evidence that female rodents exhibit cognitive resilience to chronic stress was consistently substantiated by other studies, which reported an optimal memory performance of only female stressed rats assessed in non-spatial recognition memory tasks (Luine et al., 2017; Bowman and Kelly, 2012; Wei et al., 2014).

The effects of acute stress on recognition memory are also controversial. Acute stress can indeed impair, enhance or have no effects on recognition memory performance. The heterogeneity of these effects may depend on manipulable factors such as type of stressor, type of the memory task, timing and duration of the stressor (Cazakoff et al., 2010). Male rats exposed to acute stress (inescapable restraint-tailstocks) before the sample trial of an object recognition memory task, exhibited an impaired object recognition memory (Baker and Kim, 2002). This cognitive impairment was specifically found with a 3-h delay but not with a 5-min delay between the sample and test trial of the task. Considering the effects of acute stress on recognition memory consolidation, a preclinical study reports a long-term object recognition memory impairment in rats exposed to elevated platform stress immediately after the sample trial (Maroun and Akirav, 2008). The same study reports that a different group of rats acutely stressed 3-h after the sample trial, did not exhibit recognition memory impairment. Regarding the type of stressor, restraint stress is an easy-to-use widely recognized stressor capable of inducing strong neuroendocrine and behavioral alterations, including memory impairment (Buynitsky and Mostofsky, 2009; Torrisi et al., 2021). Li and colleagues successfully utilized restraint stress to investigate the impact of acute stress on various forms of recognition memory (Li et al., 2012). They interestingly found that acute restraint stress impaired the retrieval of either short-term (4-h delay) or long-term (24-h delay) recognition memory of male mice, tested in both the NOR and NOL tasks. They also observed that acute restraint stress blocked the consolidation of short-term recognition memory into long-term memory irrespective of the task used. This detrimental effect of restraint stress on consolidation of recognition memory was further reported in a similar research article (Guercio et al., 2014).

At the mechanistic level, the study of the mechanisms underlying the effects of acute stress on recognition memory has been the main focus of a plethora of studies. There is considerable evidence indicating an overriding role of the glutamatergic neurotransmission on the mixed effects of acute stress on recognition memory (Cazakoff et al., 2010; Howland and Cazakoff, 2010). In this respect, the physiological encoding, consolidation, and retrieval of recognition memory depends on the activation of ionotropic glutamate receptors (Winters et al., 2008), within specific brain regions such as the dorsal hippocampus (dHPC) (Tuscher et al., 2018). In particular, activation of hippocampal N-methyl-D-aspartate (NMDA) receptors has been shown to be essential for the induction of the most common forms of synaptic plasticity such as long-term potentiation and long-term depression, which in turn modulate recognition memory-related processes (Warburton et al., 2013). There is also evidence that NMDA receptors in the medial prefrontal cortex-HPC pathway are involved in the encoding of the associative memory for object and place, whereas the retrieval of this type of memory relies on α-amino-3-hydroxy-5-methyl-4-isoxazolepropionic acid (AMPA) receptors-mediated neurotransmission (Chao et al., 2022). Multiple lines of evidence reports that acute stress interrupts hippocampal-dependent memory by boosting the extracellular release of glutamate (Musazzi et al., 2022), which in turn overstimulates GluN2B-containing NMDA receptors (Howland and Cazakoff, 2010; Howland and Wang, 2008). There is also evidence for an involvement of AMPA-mediated neurotransmission in the effects of acute stress on spatial recognition memory (Aguayo et al., 2018). However, it is important to underline that most of the studies evaluating the influence of acute stress on recognition memory have been performed by using exclusively male rodents. In the present study, we tested the hypothesis that acute stress could affect the consolidation of different forms of short- and long-term recognition memory in a sex-dependent manner. For this purpose, male and female C57BL6/J mice were exposed to acute restraint stress, immediately after the training session of both the novel object recognition (NOR) test and novel object location (NOL) tasks. To further test the hypothesis that post training acute stress could induce sex-specific transcriptional changes of ionotropic glutamate receptor subunits, we assessed the mRNA expression of NMDA (GluN1, GluN2A, GluN2B) and AMPA (GluA1, GluA2) subunits in the dorsal hippocampus (dHPC) at different time points. Among all NMDA/AMPA subunits, the specific subunits we selected are widely expressed in the brain and play a predominant role in synaptic plasticity mechanisms underlying learning and memory (Yashiro and Philpot, 2008).

## 2 Materials and methods

### 2.1 Animals

Male and female C57BL6/J mice (10–16 weeks old at the beginning of the experiments, Charles River Laboratories Italia, Italy) were group-housed 3–5 per cage under controlled conditions (12-h light/dark cycle, 22 ± 2 °C, food and water ad libitum) and weighed once a week until the end of each experimental protocol. The experimenter handled animals on alternate days during the week preceding the stress procedure. Animals were acclimatized to the testing room 1 h before the beginning of the tests. All experiments were carried out according to EU Directive 2010/63/EU, the Institutional Animal Care and Use Committees of Catania and the Italian Ministry of Health (authorization n.497/2022 PR).

### 2.2 Acute restraint stress

Acute restraint stress was performed as previously reported (Velli et al., 2022). Restraint-stressed (RS) mice were gently put in Falcon 50 mL conical centrifuge tubes for 2 h. During the procedure, a correct air flowing was allowed by 3 holes (0.5 mm of diameter), drilled along the sidewall and at the end of the tube. A sufficient quantity of paper towel was inserted into each tube to fill the space between the mouse and the cap. One hole was also drilled in each cup to keep the tails of the mice out of the tube. About 5 tubes containing mice, randomly chosen, were placed in conventional cages (Tecniplast, 425 x 266 × 185 mm) with a level of illumination of 400 lux. During the restraint, mice had no access to food and water. At the end of the restraint, mice were immediately put back to their home cages, with free access to food and water. During restraint, control (C) mice remained in their home cages in a different room.

### 2.3 Behavioral testing

Behavioral experiments were recorded (Sony Videocam PJ330E) and analyzed by two expert researchers. The exploration of the objects was manually scored by the researchers, while the total distance travelled during the test sessions was measured by using the ANY-maze software. Regarding the scores obtained by the two researchers, the final data derive from the mean of six measurements (three measurements for each researcher) performed by the two researchers for each animal tested. Each open field was cleaned with a 20% ethanol solution in between each test to prevent olfactory cues. We used a 12 light/12 dark cycle with lights on at 07:00 a.m. All behavioral experiments were carried out during the light phase (9.00 a.m.–4.00 p.m.). Male and female mice were stressed and tested in different weeks to minimize the influence of olfactory cues.

#### 2.3.1 Novel object location (NOL) test

The NOL test was carried out as previously described with minor modifications (Li et al., 2012). The test was performed in evenly illuminated (40 ± 1 lux) grey open fields (44 x 44 × 40 cm, Ugo Basile, Gemonio, Italy). The objects used, which were different in shape, color and size (4 x 4 × 4 cm to 6 x 6 × 6 cm), were fixed to the floor of the apparatus to avoid displacements during the test. The behavioral test started after 1 week of handling. A 2-day pretest procedure was carried out to acclimatize mice to the apparatus as well as to prevent neophobia during the test. On day 1, mice were placed into the empty apparatus and allowed to freely explore for 15 min. On day 2, mice were instead allowed to explore the apparatus containing two objects (different from those eventually used during the test) for 10 min. The objects were located in two corners of the apparatus, 10 cm from the side walls. The test consisted of one training session and one test session interspersed with 4-h or 24-h delays. During the training session (day 3), animals were placed in the center of the apparatus and then allowed to explore two copies of an identical object for a total of 10 min. During the test session (day 3 or day 4 according to the delay), mice were placed again in the center of the apparatus and allowed to explore two copies of the familiar objects explored in the training session but with a different location. One of these objects was placed in the same position occupied in the training session, while the other object (displaced object) was placed in a new location in the opposite side of the apparatus (the two objects were diagonal from each other). Exploratory behavior was defined as the mouse directing its nose toward the object at a distance of ≤2 cm. Looking around while sitting, climbing the objects and rearing against the objects were not considered exploratory behavior. Mice that failed to complete a minimum of 5 seconds (s) of total exploration in each session of the task were excluded from the analysis. If the object location memory is intact, animals should explore more the displaced object instead of the familiar one. Objects and locations were counterbalanced between animals. Discrimination between the objects during the test session was calculated using a discrimination index (DI), obtained through the following formula: [(time spent exploring the displaced object – time spent exploring the familiar object)/total exploration time]. The higher the DI, the better the cognitive performance is. The total exploration as well as the percentage of exploration of each object during the test session were further calculated.

#### 2.3.2 Novel object recognition (NOR) test

The behavioral procedure used for NOR test was similar to that of the NOL test except for the test session. During the test session, which was performed as for the NOR test after a 4-h or a 24-h delay, mice were allowed to explore for 10-min a copy of the familiar object previously explored in the training session and a novel object never encountered. Even in this case, mice with a total exploration below 5 s were excluded from the analysis. If novel object recognition memory is intact, mice normally explore more the novel object. Cognitive performance during the test session was primarily showed using the DI, obtained through the following formula: [(time spent exploring the novel object – time spent exploring the familiar object)/total exploration time. Total exploration and the percentage of exploration of each object during the test session were quantified.

### 2.4 Experimental design

The effect of acute restraint stress on consolidation of different forms of recognition memory (object location and novel object recognition), was evaluated in mice of both sexes. For this purpose, mice were exposed to 2-h restraint stress immediately after the training sessions of both the NOL and NOR tasks. To assess the effect of acute stress on either short-term or long-term memory consolidation, two different delays (4-h and 24h-delay) were used between the training session and the test session of both tasks. Different timepoints (30 min after the test sessions of the task and immediately after the end of restraint stress) were also chosen to investigate the impact of 2-h restraint stress on NMDA/AMPA receptor subunits mRNA expression.

#### 2.4.1 Experiment 1: effect of acute stress on short-term recognition memory

**Fig. 1:**
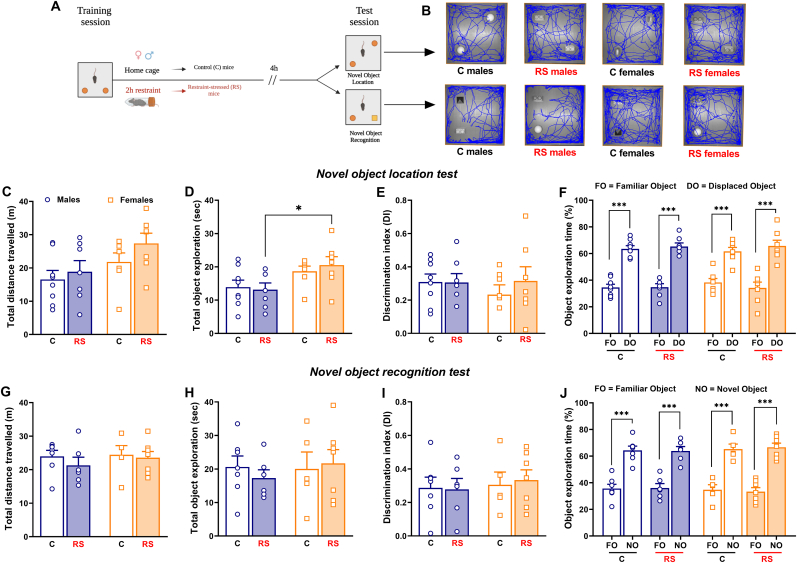
*Post training acute stress did not affect short-term object location and object recognition memory in both sexes.***(A)** Experimental procedure conceived to assess the effect of post training acute stress on the short-term (4-h delay) object location and object recognition memory in mice of both sexes, tested in the novel object location (NOL) and novel object recognition (NOR) tasks. **(B)** ANY-maze images showing the total distance travelled during the tasks. **(C)** Total distance travelled by control (C) male mice (N = 8), restraint-stressed (RS) male mice (N = 7), C female mice (N = 7) and RS female mice (N = 7) during the test session of the NOL task. **(D)** Total exploration of the two objects during the test session of the NOL task. **(E)** Discrimination index (DI) and **(F)** exploration time (%) of familiar object (FO) and displaced object (DO) obtained to evaluate the cognitive performance of mice during the test session of the NOL task. **(G)** Total distance travelled by C male mice (N = 7), RS male mice (N = 6), C female mice (N = 5) and RS female mice (N = 7) during the test session of the NOR task. **(H)** Total exploration of the two objects during the test session of the NOR task. **(I)** DI and **(J)** exploration time (%) of FO and novel object (NO) calculated to evaluate the cognitive performance of mice during the test session of the NOR task. Two-way or three-way ANOVA followed by Bonferroni post hoc test: *p < 0.05 and ***p < 0.001. Values are expressed as means ± s.e.m.

Female and male mice underwent 2-h restraint stress immediately after the training session of both the NOL and NOR tasks. In this experiment there was a 4-h delay between the training session and the test session (Fig. 1A). 3 mice were excluded because they explored the objects less than 5 s.

#### 2.4.2 Experiment 2: effect of acute stress on consolidation of long-term recognition memory and NMDA/AMPA receptor subunits mRNA expression in the dHPC

**Fig. 2:**
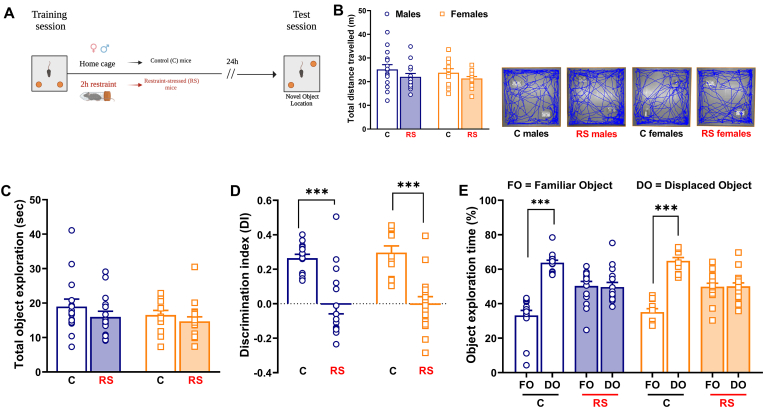
*Post training acute stress impaired long-term object location memory in both sexes.* (**A)** Experimental procedure conceived to assess the effect of post training acute stress on long-term (24-h delay) object location memory in male and female mice, tested in the novel object location (NOL) task. **(B)** Total distance travelled (with ANY-maze images) by control (C) male mice (N = 15), restraint-stressed (RS) male mice (N = 15), C female mice (N = 13) and RS female mice (N = 17) during the test session of the NOL task. **(C)** Total exploration of the two objects during the test session of the NOL task. **(D)** Discrimination index (DI) and **(E)** exploration time (%) of familiar object (FO) and displaced object (DO) calculated to assess the cognitive performance of mice during the test session of the NOL task. Two-way or three-way ANOVA followed by Bonferroni post hoc test: ***p < 0.001. Values are expressed as means ± s.e.m.

**Fig. 3:**
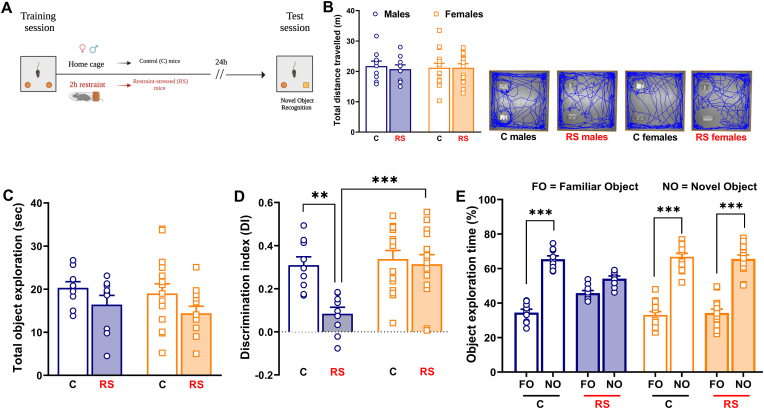
*Post training acute stress impaired long-term object recognition memory exclusively in male mice*. **(A)** Experimental procedure conceived to evaluate the effect of post training acute stress on long-term (24-h delay) object recognition memory in male and female mice, tested in the novel object recognition (NOR) task. **(B)** Total distance travelled (with ANY-maze images) by control (C) male mice (N = 9), restraint-stressed (RS) male mice (N = 9), C female mice (N = 15) and RS female mice (N = 14) during the test session of the NOR task. **(C)** Total exploration of the two objects during the test session of the NOR task. **(D)** Discrimination index (DI) and **(E)** exploration time (%) of familiar object (FO) and novel object (NO) calculated to evaluate the cognitive performance of mice during the test session of the NOR task. Two-way or three-way ANOVA followed by Bonferroni post hoc test: **p < 0.01 and ***p < 0.001. Values are expressed as means ± s.e.m.

The behavioral and stressful procedures of experiment 2 were identical to those of experiment 1 except for the delay between the training session and the test session, which was 24-h (Fig. 2A; Fig. 3A). 7 mice were excluded because they explored the objects less than 5 s.

**Fig. 4:**
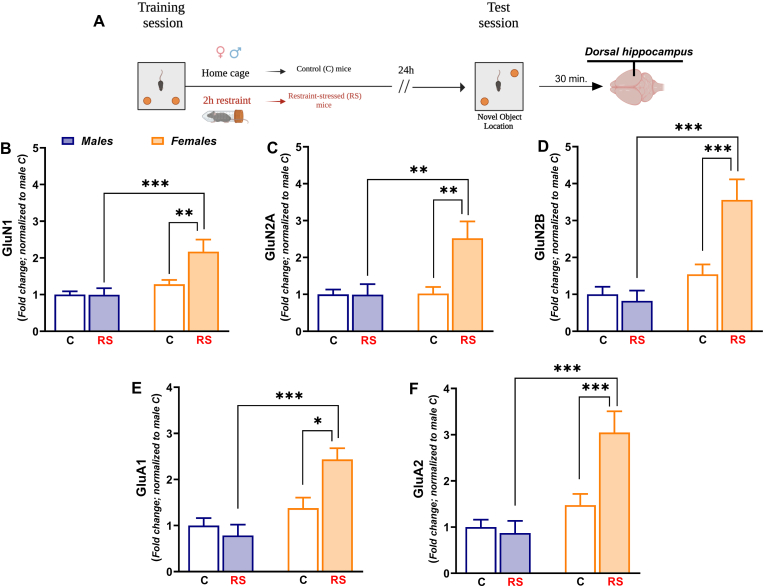
*NMDA and AMPA receptor subunits expression was significantly increased only in the dorsal hippocampus (dHPC) of restraint-stressed female mice.***(A)** Timeline: 30 min after the end of the NOL test session, control (C) male mice (n = 8), restraint-stressed (RS) male mice (N = 6), C female mice (N = 6) and RS female mice (N = 6) were sacrificed to microdissect dHPC. **(B) GluN1** mRNA expression, **(C) GluN2A** mRNA expression, **(D) GluN2B** mRNA expression, **(E) GluA1** mRNA expression and **(F) GluA2** mRNA expression. Mean fold changes are expressed relative to transcript levels of C male mice. Two-way ANOVA followed by Bonferroni post hoc test: *p < 0.05, **p < 0.01 and ***p < 0.001. Values are expressed as means ± s.e.m.

**Fig. 5:**
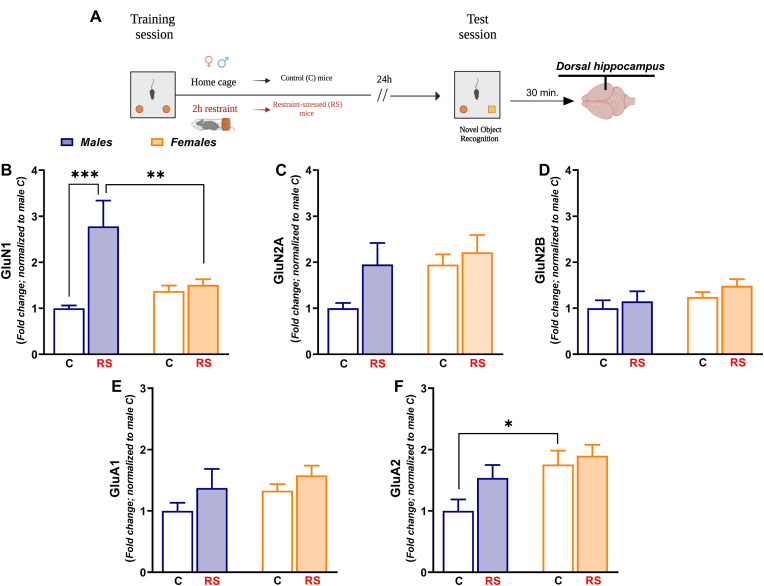
*The mRNA expression of GluN1 receptor subunit was significantly increased only in the dorsal hippocampus (dHPC) of restraint-stressed male mice after the NOR test.* (**A)** Timeline: 30 min after the end of the NOR test session, control (C) male mice (n = 6), restraint-stressed (RS) male mice (N = 7), C female mice (N = 7) and RS female mice (N = 8) were sacrificed to microdissect dHPC. **(B) GluN1** mRNA expression, **(C) GluN2A** mRNA expression, **(D) GluN2B** mRNA expression, **(E) GluA1** mRNA expression and **(F) GluA2** mRNA expression. Mean fold changes are expressed relative to transcript levels of C male mice. Two-way ANOVA followed by Bonferroni post hoc test: *p < 0.05, **p < 0.01 and ***p < 0.001. Values are expressed as means ± s.e.m.

Regarding the evaluation of NMDA/AMPA receptor subunits mRNA expression, male and female mice were sacrificed 30 min after the end of the test sessions (Fig. 4A; Fig. 5A). In this respect, synaptic plasticity and memory-related changes in the mRNA expression of NMDA and AMPA receptor subunits are generally evident after this time point (Baez et al., 2018).

#### 2.4.3 Experiment 3: assessment of NMDA/AMPA receptor subunits mRNA expression in the dHPC immediately after the end of acute stress

**Fig. 6:**
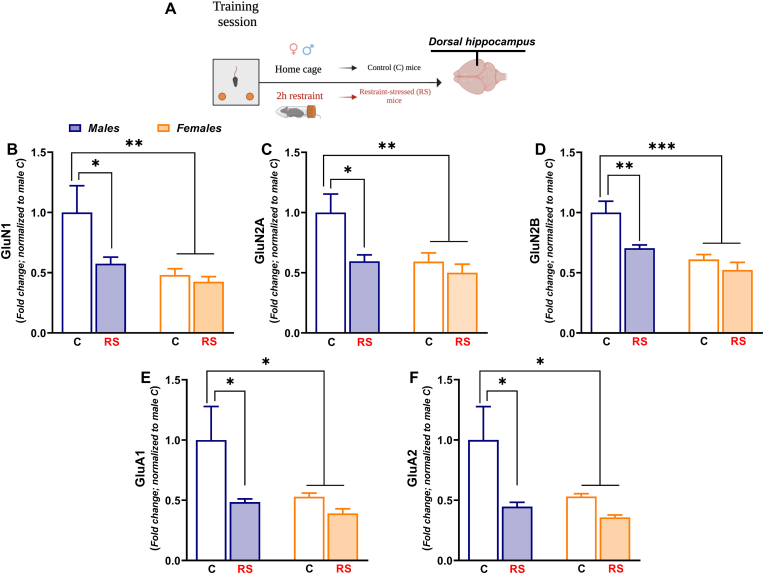
*Post training acute stress triggered immediate sex-dependent transcriptional changes of NMDA/AMPA receptor subunits in the dHPC.***(A)** Timeline: Immediately after the end of restraint stress exposure, control (C) male mice (N = 8), restraint-stressed (RS) male mice (N = 8), C female mice (N = 9) and RS female mice (N = 8) were sacrificed to microdissect dHPC. **(B) GluN1** mRNA expression, **(C) GluN2A** mRNA expression, **(D) GluN2B** mRNA expression, **(E) GluA1** mRNA expression and **(F) GluA2** mRNA expression. Mean fold changes are expressed relative to transcript levels of C male mice. Two-way ANOVA followed by Bonferroni post hoc test: *p < 0.05, **p < 0.01 and ***p < 0.001. Values are expressed as means ± s.e.m.

The behavioral and stressful procedures of experiment 3 were identical to those of experiment 1–2 except for the fact that in this experiment male and female mice were sacrificed immediately after the restraint stress (Fig. 6A). This was carried out to detect the immediate acute stress-induced changes in NMDA and AMPA receptor subunits expression, which have been reported to be rapidly detectable (Fumagalli et al., 2011; Whitehead et al., 2013).

### 2.5 Analysis of gene expression by real-time PCR

Mice were killed via cervical dislocation immediately after the end of the restraint stress or 30 min after the end of NOR or NOL test sessions. The dHPC was microdissected, flash frozen in liquid nitrogen, and then stored at −80 °C till use.

Total RNA was extracted with TRIzol reagent (Invitrogen Life Technologies, Carlsbad, CA, USA) according to the manufacturer's instructions. Total RNA extracted was resuspended in 25 μl of RNase-free water. The optical density at 260 and 280 nm was assessed using Nanodrop ND-1000 to evaluate the RNA concentration and purity. According to manufacturer's instruction, total RNA (2 μg) was converted to first-strand cDNA in a 20 μl reaction volume with 200 U of SuperScript IV, 50 ng/μl of random hexamer primers, 10 mM dNTP mix and100 mM dithiothreitol (Invitrogen Life Technologies, cat. 18091050). Reactions were carried out at 50 °C for 10 min and stopped by heating at 80 °C for 10 min. Aliquots of 100 ng of cDNA were amplified in parallel reactions using specific primer pairs for the Grin2A(Fw: 5′-ATTCAACCAGAGGGGCGTAG-3’; Rv: 5′-CCGGCCTTGTAGTTCAAGACA-3′), Grin2B (Fw: 5′-CAGTGTCATGGTATCACGCAGC-3’; Rv: 5′-ACAGCAGAGACAATGAGCAGCA-3′), Grin1 (Fw: 5′-AACGCCATACAGATGGCCCT-3’; Rv: 5′-TGGACATTCGGGTAGTCAGC-3′), Gria1 (Fw: 5′-TCCCCAACAATATCCAGATAGGG-3’; Rv: 5′-AAGCCGCATGTTCCTGTGATT-3′) and Gria2 (Fw: 5′-AATGGACGTGTTATGACTCCAGA-3’; Rv: 5′-CTGACATTCATTCCCATGCCA-3′). GAPDH was used as the reference housekeeping gene (Fw: 5′- AGGTCGGTGTGAACGGATTTG-3’; Rv: 5′- TGTAGACCATGTAGTTGAGGTCA-3′). Each qPCR (20-μl final volume) contained 0.4 μM primers, was carried out using iTaq Universal SYBR Green Supermix, according to manufacturer's instructions (Bio-Rad, cat. 1725124). Amplifications were carried out in a Applied Biosystem 7300 instrument (ThermoFisher). Each sample was run in triplicate and quantification was obtained by the 2^−^^Δ^Δ^^^*CT*^ method (Maurel et al., 2021).

### 2.6 Statistical analysis

Sample size was determined by using power analysis and was thus similar to that of previous studies (Gulisano et al., 2019). Data were analyzed using GraphPad Prism 8 (GraphPad Software, La Jolla, CA, USA). The D'Agostino-Pearson omnibus normality test was carried out to assess data distribution. The Levene's test was also used to verify equality of variances. Because all data assumed a normal distribution, data were analyzed by using parametric tests (two-way ANOVA or three-way ANOVA). For all data analyses, upon confirmation of significant main effects, differences among individual means were evaluated using Bonferroni multiple comparisons test. P values < 0.05 were considered significant. Details concerning ANOVA (main effects, interactions) are also reported in the supplementary data Table S1. All data are presented as means ± s. e. m.

## 3 Results

### 3.1 Experiment 1: Acute stress did not impair short-term object location memory and object recognition memory in both sexes

During the test session of the NOL test, RS female mice were significantly more active than male mice [Fig. 1B and C *Sex*: F_(1, 25)_ = 5.421; P = 0.0283]. Regarding the total exploration of the objects during the test session of the NOL test, RS female mice significantly spent more time exploring the two objects compared to RS male mice [Fig. 1D; *Sex*: F_(1, 25)_ = 8.524; P = 0.0073]. Interestingly, acute stress did not affect the ability of RS male and female mice to discriminate between the displaced object and the familiar object, calculated as DI (Fig. 1E), after a 4-h delay between the training session and the test phase. Indeed, RS male and female mice, as C mice, significantly explored more the displaced object [Fig. 1F; *Object*: F _(1, 50)_ = 169.1; P < 0.0001]. There were no significant differences between male and females in the total exploration of the objects during the training session of the NOL test (Fig. S1A).

There were no differences among groups in the total distance travelled as well as in the total exploration of the objects during the test session of the NOR test (Fig. 1G and H). Considering the DI analysis, neither the cognitive performance of RS male and female mice during the NOR test was affected after a 4-h delay between the training session and the test phase (Fig. 1I). RS mice of both sexes significantly spent more time exploring the novel object as their respective C mice [Fig. 1J; *Object*: F _(1, 42)_ = 163.2; P < 0.0001]. There were no significant differences between male and females in the total exploration of the objects during the training session of the NOR test (Fig. S1B).

### 3.2 Experiment 2: Acute stress altered long-term object recognition memory but not object location memory in a sex-dependent manner

No significant differences were found among the groups in the total distance travelled and in the total exploration of the objects during the test session of the NOL test (Fig. 2B and C), which was performed 24-h after the training session to assess long-term object location memory (24-h delay). Interestingly, acute stress disrupted the long-term object location memory in both sexes, as indicated by the significant lower DI of RS male and female mice in comparison with their respective C mice [Fig. 2D; *Stress*: F_(1, 56)_ = 50.08; P < 0.0001]. In fact, RS mice of both sexes failed to recognize and discriminate the familiar object from the displaced one spending approximatively the same amount of time exploring both objects [Fig. 2E; *Object*: F _(1, 112)_ = 90.16; P < 0.0001. *Object x Stress*: F_(1, 112)_ = 92.49; P < 0.0001]. Male and female mice spent the same amount of time exploring the two objects during the training session of the NOL test (Fig. S1C).

In line with the previous NOL results, we did not find significant differences among the four groups with respect to the total distance travelled and the total exploration of the objects in the test session of the NOR test (Fig. 3B and C). Also for the NOR test, mice performed the test session 24-h after the training session in order to evaluate their long-term object recognition memory (24-h delay). We intriguingly found a sex-dependent effect of acute stress on object recognition memory. RS male mice were indeed significantly impaired in the NOR test, as indicated by their lower DI compared to that of C male mice [Fig. 3D; *Stress*: F_(1, 43)_ = 8.590; P = 0.0054. *Sex*: F_(1, 43)_ = 9.207; P = 0.0041. *Stress x Sex*: F _(1, 43)_ = 5.586; P = 0.0227]. RS male mice spent roughly the same amount of time exploring both the familiar and the novel objects [Fig. 3E; *Object*: F_(1, 86)_ = 301.6; P < 0.0001. *Object x Stress*: F_(1, 86)_ = 17.18; P < 0.0001. *Object x Sex:* F_(1, 86)_ = 18.41; P < 0.0001. *Object x Sex x Stress*: F_(1, 86)_ = 11.17; P = 0.0012]. In contrast, RS female mice were unimpaired in the NOR test, as showed by the DI similar to those of C female mice (Fig. 3D). RS female mice were capable to discriminate between the two objects exploring significantly more the novel object (Fig. 3E). During the training session of the NOR test, male and female mice similarly explored the two objects (Fig. S1D).

### 3.3 Experiment 2: Post training acute stress induced sex- and type of memory-dependent transcriptional changes of NMDA/AMPA receptor subunits in the dHPC

Acute stress induced sex-dependent changes of NMDA/AMPA receptor subunits expression in the dHPC, 30 min after the end of the test session of the NOL test. Notably, although acute stress equally disrupted the object location memory of both RS male and female mice, the mRNA expression of **GluN1** [Fig. 4B; *Stress*: F_(1, 22)_ = 5.489; P = 0.0286. *Sex*: F_(1, 22)_ = 14.87; P = 0.0009. *Stress x Sex*: F_(1, 22)_ = 5.598; P = 0.0272], **GluN2A** [Fig. 4C; *Stress*: F_(1, 22)_ = 7.394; P = 0.0125. *Sex*: F_(1, 22)_ = 7.976; P = 0.0099. *Stress x Sex*: F_(1, 22)_ = 7.545; P = 0.0118] and **GluN2B** [Fig. 4D; *Stress*: F_(1, 22)_ = 7.396; P = 0.0125. *Sex*: F_(1, 22)_ = 23.44; P < 0.0001. *Stress x Sex*: F_(1, 22)_ = 10.49; P = 0.0038], was significantly increased exclusively in the dHPC of RS female mice. Similarly, mRNA expression of **GluA1** [Fig. 4E; *Sex* F_(1, 22)_ = 22.49; P < 0.0001. *Stress x Sex*: F_(1, 22)_ = 8.866; P = 0.0069] and **GluA2** [Fig. 4F; *Stress:* F _(1, 22)_ = 6.347; P = 0.0195. *Sex*: F_(1, 22)_ = 21.43; P = 0.0001. *Stress x Sex*: F _(1, 22)_ = 8.828; P = 0.0070] was increased only in the dHPC of RS female mice.

Acute stress also triggered divergent sex-dependent changes of NMDA/AMPA receptor subunits expression in the dHPC, 30 min after the end of the test session of the NOR test. A significant increase of **GluN1** mRNA expression was uncovered only in the dHPC of RS male mice [Fig. 5B; *Stress*: F _(1, 24)_ = 10.33; P = 0.0037. *Stress x Sex*: F _(1, 24)_ = 7.666; P = 0.0107], while the mRNA expression of **GluN2A** and **GluN2B** were not significantly changed (Fig. 5C and D). Regarding AMPA receptor subunits, there was no difference in the mRNA expression of **GluA1** (Fig. 5E). Noteworthy, we found a sex-dependent but not stress-induced change in the mRNA expression of **GluA2**. The mRNA expression of GluA2 was significantly higher in the dHPC of C female mice in comparison with C male mice [Fig. 5F; *Sex*: F _(1, 24)_ = 7.590; P = 0.0110].

### 3.4 Experiment 3: Post training acute stress induced rapid sex-dependent transcriptional changes of NMDA/AMPA receptor subunits in the dHPC

The assessment of NMDA/AMPA receptor subunits mRNA expression immediately after the end of 2h-restraint stress, revealed immediate and marked sex-dependent transcriptional changes in the dHPC. The mRNA expression of **GluN1** [Fig. 6B; *Stress*: F _(1, 29)_ = 4.308; P = 0.0469. *Sex*: F_(1, 29)_ = 8.304; P = 0.0074], **GluN2A** [Fig. 6C; *Stress*: F_(1, 29)_ = 6.915; P = 0.0135. *Sex*: F _(1, 29)_ = 7.049; P = 0.0127] and **GluN2B** [Fig. 6D; *Stress*: F _(1, 29)_ = 10.08; P = 0.0035. *Sex*: F _(1, 29)_ = 22.20; P < 0.0001], was significantly decreased in the dHPC of RS male mice compared to C male mice. Notably, a significant decrease of all NMDA subunits mRNA expression was found in the dHPC of C and RS female mice compared to C male mice (Fig. 6B–D). An overlapping pattern of significant decrease in mRNA expression of **GluA1** [Fig. 6E; *Stress:* F_(1, 29)_ = 5.616; P = 0.0247. *Sex* F_(1, 29)_ = 4.187; P = 0.0499] and **GluA2** [Fig. 6F; *Stress:* F_(1, 29)_ = 7.124; P = 0.0123. *Sex*: F_(1, 29)_ = 4.194; P = 0.0497] was found in the dHPC of RS male mice, C and RS female mice in comparison with C male mice.

## 4 Discussion

This study indicates that the effects of acute stress on recognition memory can be strongly influenced by multiple biological variables including sex. This study further suggests that the same stress-induced memory impairment in both sexes can be triggered by different sex-dependent molecular mechanisms.

The lack of effect of acute stress on short-term object location and object recognition memory is consistent with previous reports (Li et al., 2012; Shields et al., 2017). It is important in this respect to remark that the timing of the stressor and the delay between the stressor and the test are important factors influencing the impact of stress on different types of recognition memory. It is further important to underline that changing the duration of the acute restraint stress, which in this study was longer than previous studies (Li et al., 2012; Guercio et al., 2014), did not influence the effect of stress on short-term recognition memory. The fact that RS female mice showed higher locomotor activity and exploratory behavior compared to RS male mice during the training session of the NOL test (4-h delay), corroborates previous studies showing similar results (Gupta and Chattarji, 2021; Lipatova et al., 2018; van Haaren et al., 1990). These specific sex differences may be also related to the interindividual differences in stress sensitivity because we did not find sex differences in the other experiments performed in this study. It is however important to remark that these sex differences did not affect the cognitive performance.

Our findings reveal that acute stress equally disrupted the consolidation of long-term (24h-delay) object location memory in both sexes. Whereas the detrimental effect of acute stress on the consolidation of object location memory in male mice is in line with previous findings (Li et al., 2012), here we report for what is to our knowledge the first time the same detrimental effect on female mice. The few studies that have investigated possible sex differences in the effect of stress on recognition memory, have been carried out using chronic stress procedures (Luine et al., 2017). One of this studies reports cognitive resilience of female rats exposed to chronic stress and assessed in both spatial and recognition memory tasks (Bowman and Kelly, 2012). Our findings thus demonstrate that acute stress can induce effects on spatial recognition memory that do not resemble those triggered by chronic stress.

We unraveled sex differences in the effects of acute stress on the consolidation of long-term object recognition memory. Also in this case, the acute stress-induced impairment in object recognition memory observed in male mice, supports previous reports showing similar results (Li et al., 2012; Guercio et al., 2014). This evidence is however not in line with other results reporting no effects of acute stress on the consolidation of object recognition memory of rats (Maroun and Akirav, 2008). In contrast, we provide evidence that acute stress did not impair the consolidation of long-term object recognition memory in female mice. This evidence corroborates the concept of female cognitive resilience reported in previous works examining the impact of chronic stress on recognition memory (Luine et al., 2017).

At the mechanistic level, how acute stress impacts long-term but not short-term recognition memory needs further investigation. It is important to underline that the neural mechanisms subserving short-term memories are basically different from those subserving long-term memories (Moore et al., 2013). In this scenario, the glutamate system plays a key role. Indeed, it is well-known that acute stress induces a rapid and sustained increase of glutamate release in several brain regions, including the prefrontal cortex and HPC (Popoli et al., 2011). This glutamate release leads to an increased basal glutamatergic transmission driven by an augmented surface expression of NMDA and AMPA receptors at the postsynaptic plasma membrane (Sanacora et al., 2022). It is also well-documented that overactivation of NMDA receptors can trigger excitotoxicity, impair synaptic plasticity and produces neurodegeneration (Papouin et al., 2012). However, these NMDA receptor-mediated detrimental effects are triggered only after a long-term coactivation of both synaptic and extrasynaptic NMDA receptors (Zhou et al., 2013). By contrast, a short-term coactivation of both synaptic and extrasynaptic NMDA receptors promotes pro-survival cell signaling (Zhou et al., 2013). Thus, the evidence that acute stress affects long-term but not short-term recognition memory might be related to these time-dependent detrimental effects induced by the overstimulation of NMDA receptors, which ultimately may interrupt the neural mechanisms underlying the consolidation of long-term memory. In line with this, here we found that acute stress triggered sex-, time- and memory-dependent transcriptional changes of NMDA and AMPA receptor subunits in the dHPC. Remarkably, in spite of the same acute stress-induced long-term object location memory impairment found in both sexes, a robust increase in the mRNA expression of all NMDA and AMPA receptor subunits was evident only in the dHPC of RS female mice. In this respect the few available data indicate that females have an increased glutamatergic neurotransmission compared to males and especially an increased AMPA/NMDA receptor-mediated neurotransmission (Wickens et al., 2018). Under basal conditions, higher AMPA receptor-mediate synaptic responses have been recorded in hippocampal slices of female rats compared to male rats (Monfort et al., 2015). Female rodents also appear to be more sensitive to pharmacological blockade of NMDA receptors. It has been shown indeed a more sensitivity to MK-801-induced excitotoxic damage (Wozniak et al., 1998) as well as stronger behavioral responses to ketamine in female rats (McDougall et al., 2017). These sex differences in the sensitivity of the glutamate system might therefore explain the increase NMDA and AMPA receptor subunits mRNA expression discovered only in the dHPC of RS female mice. Thus, our data suggest that acute stress may induce a more pronounced dysfunction of the glutamatergic neurotransmission in the dHPC of female mice.

On the contrary, after the NOR test session, an increase in the mRNA expression of only the GluN1 subunit was uncovered only in the dHPC of RS male mice, which exhibited long-term object recognition memory impairment. This indicates that the molecular and behavioral effects of acute stress can be biased by multifactorial interactions, such as sex x type of memory interaction. However, studies investigating the neural substrates underlying object recognition memory have provided controversial results (Howland and Wang, 2008); besides the dHPC, other brain regions such as the perirhinal cortex might play a more important role in this scenario (Warburton et al., 2013). Moreover, the exclusive stress-induced increase of the obligatory subunit GluN1 might be a maladaptive response already found in several brain regions, including the hippocampus, of stressed male rodents (Nasca et al., 2015; Schwendt and Jezova, 2001). Notably, we found that after the NOR test the mRNA expression of GluA2 was significantly higher in the dHPC of C female mice in comparison with C male mice, while there were no differences in the mRNA expression of GluA1. These results are in line with a previous study showing basal sex differences in the composition of AMPA receptors. In particular, this study reports no sex differences in the amount of GluA1 but a higher amount of the GluA2 subunit in the HPC of female rats (Monfort et al., 2015).

We intriguingly further found sex-dependent transcriptional changes of NMDA/AMPA receptor subunits in the dHPC immediately after the end of the restraint stress. In line with previous findings (Baker and Kim, 2002), the significant decrease in the mRNA expression of NMDA and AMPA receptor subunits observed in RS male mice might contribute in the induction of synaptic plasticity dysfunctions, which in turn might be responsible for the memory impairment observed with a long delay. The significant decrease in the mRNA expression of NMDA and AMPA receptor subunits observed in C female mice indicates that encoding processes trigger basal sex-dependent rearrangement of the glutamate system. This is consistent with the aforementioned findings demonstrating basal sex differences in AMPA/NMDA receptor-mediated neurotransmission (Wickens et al., 2018).

We did not assess the estrous cycle of female mice throughout the experimental procedures because this was not within the scope of the present study. However, because ovarian hormones play an unquestionable central role in the neural mechanisms subserving spatial and recognition memory (Boulware et al., 2013; Orr et al., 2012; Rinaudo et al., 2022), further research is warranted in order to establish a possible involvement of the fluctuating ovarian hormones in the puzzling effects exerted by acute stress on these cognitive domains.

At the therapeutic level, this study supports the emerging view indicating sex as a fundamental biological variable that should not be overlooked in the context of personalized and targeted treatments. In this regard, the vast majority of the preclinical and clinical neuroscience/neuropharmacology studies have been performed using only male subjects (Shansky and Murphy, 2021). It is thought that this has probably contributed to higher rates of misdiagnosis and adverse drug reactions in women (Zucker and Prendergast, 2020). Our findings suggest a possible heightened NMDA and AMPA receptors sensitivity to the acute stress-induced aberrant and sustained glutamate release in the dHPC of female mice. Previous findings have also provided evidence for sex-biased functioning of druggable receptors (Doyle et al., 2017; Valentino et al., 2013).

In conclusion, more targeted pharmacological treatments may arise studying sex-dependent mechanisms, which may ultimately imply different targets and different doses to improve efficacy and decrease the risk of adverse drug reactions.

## CRediT authorship contribution statement

**Sebastiano A. Torrisi:** Conceptualization, Formal analysis, Data curation, Investigation, Methodology, Project administration, Writing – original draft, Writing – review & editing. **Silvia Rizzo:** Formal analysis, Data curation, Investigation, Methodology, Software. **Samuele Laudani:** Formal analisys, Investigation. **Alessandro Ieraci:** Formal analysis. **Filippo Drago:** Supervision, Funding acquisition. **Gian Marco Leggio:** Conceptualization, Supervision, Funding acquisition, Investigation, Methodology, Project administration, Resources, Writing – original draft, Writing – review & editing.

## Declaration of competing interest

The authors declare they have no conflict of interest.

## Data Availability

Data will be made available on request.
