# Supplementary material for: Acute stress alters recognition memory and AMPA/NMDA receptor subunits in a sex-dependent manner
Source: Neurobiol Stress. 2023 May 26;25:100545. doi: 10.1016/j.ynstr.2023.100545 (PMC10244889; doi:10.1016/j.ynstr.2023.100545)
Supplement: Multimedia component 1 [file mmc1.docx]

**SUPPLEMENTARY INFORMATION**

Torrisi et al.

**SUPPLEMENTARY FIGURE**

**Figure S1: Total exploration of the objects performed by male and female mice during the training sessions of the tasks.**

**(A)** Total exploration of the objects performed by male mice (N = 15) and female mice (N = 14) during the training session of the novel object location (NOL) task (4h-delay). **(B)** Total exploration of the objects performed by male mice (N = 13) and female mice (N = 12) during the training session of the novel object recognition (NOR) task (4h-delay). **(C)** Total exploration of the objects performed by male mice (N = 30) and female mice (N = 30) during the training session of the NOL task (24h-delay). **(D)** Total exploration of the objects performed by male mice (N = 18) and female mice (N = 29) during the training session of the NOR task (24h-delay). Values are expressed as means ± s.e.m.


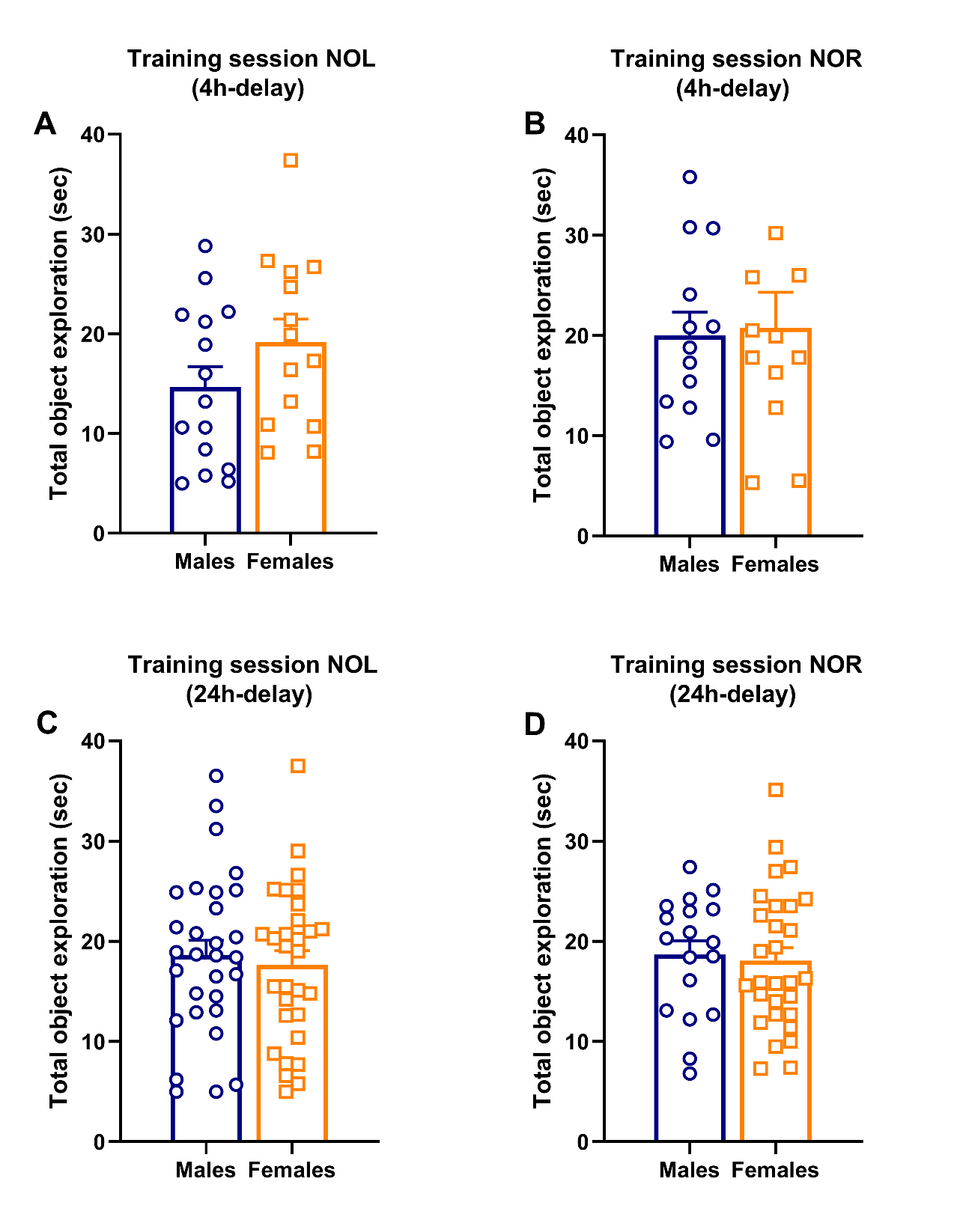


**SUPPLEMENTARY DATA**

**Table S1: Statistical Analysis Details**

| Statistical Analysis Details | | | | |  |
| --- | --- | --- | --- | --- | --- |
| **Fig. #** | **Statistical test** | **Factors, Degree of freedom & F/t value** | **p-value** | **Significance** |  |
|  |  |  |  |  |  |
| 1C | Two-way ANOVA | Sex F (1, 25) = 5.421 | **0.0283** | ***** |  |
|  |  | Stress F (1, 25) = 1.771 | 0.1953 | ns |  |
|  |  | Stress x Sex F (1, 25) = 0.3005 | 0.5884 | ns |  |
| 1D | Two-way ANOVA | Sex F (1, 25) = 8.524 | **0.0073** | ****** |  |
|  |  | Stress F (1, 25) = 0.06835 | 0.7959 | ns |  |
|  |  | Stress x Sex F (1, 25) = 0.3988 | 0.5335 | ns |  |
| 1E | Two-way ANOVA | Sex F (1, 25) = 0.2832 | 0.5993 | ns |  |
|  |  | Stress F (1, 25) = 0.3971 | 0.5343 | ns |  |
|  |  | Stress x Sex F (1, 25) = 0.4640 | 0.5020 | ns |  |
| 1F | Three-way ANOVA | Object F (1, 50) = 169.1 | **< 0.0001** | ******** |  |
|  |  | Sex F (1, 50) = 0.04698 | 0.8293 | ns |  |
|  |  | Stress F (1, 50) = 0.04698 | 0.8293 | ns |  |
|  |  | Object x Sex F (1, 50) = 0.2874 | 0.5942 | ns |  |
|  |  | Object x Stress F (1, 50) = 1.228 | 0.2730 | ns |  |
|  |  | Sex x Stress F (1, 50) = 0.04698 | 0.8293 | ns |  |
|  |  | Object x Sex x Stress F (1, 50) = 0.5581 | 0.4585 | ns |  |
| 1G | Two-way ANOVA | Sex F (1, 21) = 0.4099 | 0.6856 | ns |  |
|  |  | Stress F (1, 21) = 0.6745 | 0.4207 | ns |  |
|  |  | Stress x Sex F (1, 21) = 0.1685 | 0.6856 | ns |  |
| 1H | Two-way ANOVA | Sex F (1, 21) = 0.2499 | 0.6224 | ns |  |
|  |  | Stress F (1, 21) = 0.04724 | 0.8300 | ns |  |
|  |  | Stress x Sex F (1, 21) = 0.4311 | 0.5186 | ns |  |
| 1I | Two-way ANOVA | Sex F (1, 21) = 0.3101 | 0.5835 | ns |  |
|  |  | Stress F (1, 21) = 0.02032 | 0.8880 | ns |  |
|  |  | Stress x Sex F (1, 21) = 0.07730 | 0.7837 | ns |  |
| 1J | Three-way ANOVA | Object F (1, 42) = 163.2 | **<0.0001** | ******** |  |
|  |  | Sex F (1, 42) = 9.105e-030 | >0.9999 | ns |  |
|  |  | Stress F (1, 42) = 0.000 | >0.9999 | ns |  |
|  |  | Object x Sex F (1, 42) = 0.6203 | 0.4354 | ns |  |
|  |  | Object x Stress F (1, 42) = 0.04065 | 0.8412 | ns |  |
|  |  | Sex x Stress F (1, 42) = 0.000 | >0.9999 | ns |  |
|  |  | Object x Sex x Stress F (1, 42) = 0.1546 | 0.6962 | ns |  |
| 2B | Two-way ANOVA | Sex F (1, 56) = 0.5210 | 0.4734 | ns |  |
|  |  | Stress F (1, 56) = 3.406 | 0.0703 | ns |  |
|  |  | Stress x Sex F (1, 56) = 0.05891 | 0.8091 | ns |  |
| 2C | Two-way ANOVA | Sex F (1, 56) = 1.274 | 0.2638 | ns |  |
|  |  | Stress F (1, 56) = 2.141 | 0.1490 | ns |  |
|  |  | Stress x Sex F (1, 56) = 0.1278 | 0.7221 | ns |  |
| 2D | Two-way ANOVA | Sex F (1, 56) = 0.2603 | 0.6119 | ns |  |
|  |  | Stress F (1, 56) = 50.08 | **<0.0001** | ******** |  |
|  |  | Stress x Sex F (1, 56) = 0.09113 | 0.7639 | ns |  |
| 2E | Two-way ANOVA | Object F (1, 112) = 90.16 | **<0.0001** | ******** |  |
|  |  | Sex F (1, 112) = 0.2250 | 0.6362 | ns |  |
|  |  | Stress F (1, 112) = 0.2250 | 0.6362 | ns |  |
|  |  | Objects x Sex F (1, 112) = 2.714e-005 | 0.9959 | ns |  |
|  |  | Objects x Stress F (1, 112) = 92.49 | **<0.0001** | ******** |  |
|  |  | Sex x Stress F (1, 112) = 0.2250 | 0.6362 | ns |  |
|  |  | Objects x Sex x Stress F (1, 112) = 0.07243 | 0.7883 | ns |  |
| 3B | Two-way ANOVA | Sex F (1, 43) = 0.0004335 | 0.9835 | ns |  |
|  |  | Stress F (1, 43) = 0.1046 | 0.7480 | ns |  |
|  |  | Stress x Sex F (1, 43) = 0.1127 | 0.7387 | ns |  |
| 3C | Two-way ANOVA | Sex F (1, 43) = 0.6437 | 0.4268 | ns |  |
|  |  | Stress F (1, 43) = 4.333 | **0.0434** | ***** |  |
|  |  | Stress x Sex F (1, 43) = 0.02883 | 0.8660 | ns |  |
| 3D | Two-way ANOVA | Sex F (1, 43) = 9.207 | **0.0041** | ****** |  |
|  |  | Stress F (1, 43) = 8.590 | **0.0054** | ****** |  |
|  |  | Stress x Sex F (1, 43) = 5.586 | **0.0227** | ***** |  |
| 3E | Two-way ANOVA | Object F (1, 86) = 301.6 | **<0.0001** | ******** |  |
|  |  | Sex F F (1, 86) = 0.000 | >0.9999 | ns |  |
|  |  | Stress F (1, 86) = 7.061e-030 | >0.9999 | ns |  |
|  |  | Objects x Sex F (1, 86) = 18.41 | **<0.0001** | ******** |  |
|  |  | Objects x Stress F (1, 86) = 17.18 | **<0.0001** | ******** |  |
|  |  | Sex x Stress F (1, 86) = 8.717e-030 | >0.9999 | ns |  |
|  |  | Objects x Sex x Stress F (1, 86) = 11.17 | **0.0012** | ****** |  |
| 4B | Two-way ANOVA | Sex F (1, 22) = 14.87 | **0.0009** | ******* |  |
|  |  | Stress F (1, 22) = 5.489 | **0.0286** | ***** |  |
|  |  | Stress x Sex F (1, 22) = 5.598 | **0.0272** | ***** |  |
| 4C | Two-way ANOVA | Sex F (1, 22) = 7.976 | **0.0099** | ****** |  |
|  |  | Stress F (1, 22) = 7.394 | **0.0125** | ***** |  |
|  |  | Stress x Sex F (1, 22) = 7.545 | **0.0118** | ***** |  |
| 4D | Two-way ANOVA | Sex F (1, 22) = 23.44 | **<0.0001** | ******** |  |
|  |  | Stress F (1, 22) = 7.396 | **0.0125** | ***** |  |
|  |  | Stress x Sex F (1, 22) = 10.49 | **0.0038** | ****** |  |
| 4E | Two-way ANOVA | Sex F (1, 22) = 22.49 | **<0.0001** | ******** |  |
|  |  | Stress F (1, 22) = 3.866 | 0.0620 | ns |  |
|  |  | Stress x Sex F (1, 22) = 8.866 | **0.0069** | ****** |  |
| 4F | Two-way ANOVA | Sex F (1, 22) = 21.43 | **0.0001** | ******* |  |
|  |  | Stress F (1, 22) = 6.347 | **0.0195** | ***** |  |
|  |  | Stress x Sex F (1, 22) = 8.828 | **0.0070** | ****** |  |
| 5B | Two-way ANOVA | Sex F (1, 24) = 2.258 | 0.1459 | ns |  |
|  |  | Stress F (1, 24) = 10.33 | **0.0037** | ****** |  |
|  |  | Stress x Sex F (1, 24) = 7.666 | **0.0107** | ***** |  |
| 5C | Two-way ANOVA | Sex F F (1, 24) = 3.129 | 0.0896 | ns |  |
|  |  | Stress F (1, 24) = 3.172 | 0.0876 | ns |  |
|  |  | Stress x Sex F (1, 24) = 0.9949 | 0.3285 | ns |  |
| 5D | Two-way ANOVA | Sex F (1, 24) = 3.088 | 0.0916 | ns |  |
|  |  | Stress F (1, 24) = 1.414 | 0.2461 | ns |  |
|  |  | Stress x Sex F (1, 24) = 0.07918 | 0.7808 | ns |  |
| 5E | Two-way ANOVA | Sex F (1, 24) = 1.847 | 0.1868 | ns |  |
|  |  | Stress F (1, 24) = 2.514 | 0.1259 | ns |  |
|  |  | Stress x Sex F (1, 24) = 0.1029 | 0.7512 | ns |  |
| 5F | Two-way ANOVA | Sex F (1, 24) = 7.590 | **0.0110** | ***** |  |
|  |  | Stress F (1, 24) = 2.788 | 0.1079 | ns |  |
|  |  | Stress x Sex F (1, 24) = 0.9382 | 0.3424 | ns |  |
| 6B | Two-way ANOVA | Sex F (1, 29) = 8.304 | **0.0074** | ****** |  |
|  |  | Stress F (1, 29) = 4.308 | **0.0469** | ***** |  |
|  |  | Stress x Sex F (1, 29) = 2.528 | 0.1227 | ns |  |
| 6C | Two-way ANOVA | Sex F (1, 29) = 7.049 | **0.0127** | ***** |  |
|  |  | Stress F (1, 29) = 6.915 | **0.0135** | ***** |  |
|  |  | Stress x Sex F (1, 29) = 2.725 | 0.1096 | ns |  |
| 6D | Two-way ANOVA | Sex F (1, 29) = 22.20 | **<0.0001** | ******** |  |
|  |  | Stress F (1, 29) = 10.08 | **0.0035** | ****** |  |
|  |  | Stress x Sex F (1, 29) = 2.980 | 0.0949 | ns |  |
| 6E | Two-way ANOVA | Sex F (1, 29) = 4.187 | **0.0499** | ***** |  |
|  |  | Stress F (1, 29) = 5.616 | **0.0247** | ***** |  |
|  |  | Stress x Sex F (1, 29) = 1.874 | 0.1816 | ns |  |
| 6F | Two-way ANOVA | Sex F (1, 29) = 4.194 | **0.0497** | ***** |  |
|  |  | Stress F (1, 29) = 7.124 | **0.0123** | ***** |  |
|  |  | Stress x Sex F (1, 29) = 1.942 | 0.1740 | ns |  |
| S1A | Unpaired t test (Two-tailed) | t=1.463 df=27 | 0.1551 | ns |  |
| S1B | Unpaired t test (Two-tailed) | t=0.1876, df=23 | 0.8528 | ns |  |
| S1C | Unpaired t test (Two-tailed) | t=0.4837, df=58 | 0.6304 | ns |  |
| S1D | Unpaired t test (Two-tailed) | t=0.3043, df=45 | 0.7623 | ns |  |
